# Notes and News

**Published:** 1898-12-24

**Authors:** 


### NOTES AND NEWS.

(COLLECTED AND COMMUNICATED.)

A FUND to endow the Dartmore Home for Crippled Boys in London is to be raised to the memory of the late General Sir Lynedoch Gardiner, Equerry to the Queen.

THROUGH the will of Miss Annie Hamilton, a Court dressmaker, University College Hospital, the Westminister Hospital, St. Thomas's, Guy's, and the London Hospital will ultimately each receive about £8,000.

THE Duchess of Devonshire recently opened the Whitworth Hospitals at Darley Dale. These hospitals were originated by the late Lady Whitworth, and the scheme completed through the will of her husband. Besides the cost of establishing the institution an endowment fund of £430 per annum has been provided.

THE new Convalescent Home for Jewish patients was consecrated by the Chief Rabbi this week. It is called the Clara Baroness de Hirsch Convalescent Home, who purchased the freehold for £16,000, and has fully endowed it. Tudor House, which has been adapted for the purposes of the home, is a fine residence at Hampstead.

THE meeting at Stafford House, under the auspices of the Duchess of Sutherland, to formulate arrangements for a bazaar in aid of Charing Cross Hospital was a complete success. It was arranged that the bazaar should be held in June, and the patronage of the Queen was announced, as well as a proposal to ask the Princess of Wales and the Duchess of Connaught to open the bazaar on the first and second days respectively.

SIR W. H. HOULDSWORTH's amendment to a resolution respecting the site of the Manchester Royal Infirmary has been carried by a large majority, a poll having been demanded. It provides that the Board of Management shall be requested to open negotiations with the Manchester Corporation with a view to disposing of the site of the present infirmary advantageously to both city and infirmary, providing an out-patient department and receiving house for the infirmary in the centre of the city.

A SEVERE epidemic of diphtheria is reported from Brighton, and an order has been given to close the schools.

THE Sea Bathing Hospital at Margate is again open. Many very necessary improvements have been effected. The drainage has been reconstructed, better quarters for nurses and servants have been secured, and two wards have been arranged for the reception of other than scrofulous cases. This is as intended in the original scheme of the hospital, which has been rechristened as above from the less pleasant and somewhat misleading title of Sea Bathing Infirmary for Scrofula. We learn that a large proportion of the expense of the alterations has been borne by one of the members of the court of directors.

THE following gentlemen have passed the requisite examination and have been duly elected Fellows of the College of Surgeons of Edinburgh: Mr. Charles Edward Douglas, M.B., C.M. and M.D.Edin., Winthank House, Cupar, Fife; Mr. Charles Wright Edwards, M.R.C.S.Eng., L.R.C.P.Lond., Sackville House, East Grinstead, Sussex; Mr. Hugh Tener Galbraith, L.R.C.S.E., &c., Durban, Natal; Mr. Carel Hendrik Kruger, M.B., C.M.Edin., 13, Glengyle Terrace, Edinburgh; and Mr. Ellis Pearson, L.R.C.S.E., &c., 46, Carlton Road, Birkenhead. The gold medal presented to the College by Colonel William Lorimer Bathgate, in memory of his late father, Mr. William M'Phune Bathgate, F.R.C.S.E., Lecturer on Materia Medica in the Extra-Academical School, has been awarded, after the annual competitive written examination in materia medica, to Mr. George Robertson, 53, High Street, Dunfermline.

THE laying of the foundation-stone of the new Jenny Lind Infirmary for Sick Children at Norwich was attended with many pleasant incidences. The ceremony was performed by Master G. Colman, a grandson of Mr. Colman, the donor of the site, and son of the chairman of the infirmary. Beneath the foundation-stone was placed a bottle, containing a copy of a local newspaper, a local time-table, a new penny, and a brass plate, on which was inscribed: "Jenny Lind Infirmary was founded in 1853. Rebuilt 1898. Foundation-stone laid by G. R. Colman. Norwich population, 113,000. Fastest train to London, 2 hours, 37 minutes. Electric tramways commenced. Price of large sheep, 63s. Bricklayer's wages, 7½d. an hour." At the invitation of the committee the eldest son and only daughter of Madame Jenny Lind were present; and the former, in addressing those present at the ceremony, stated that the establishment of the Jenny Lind Infirmary, through his mother's instrumentality, had been the forerunner of several similar institutions in Sweden. The new infirmary is intended to commemorate the Queen's Jubilee, and for this purpose the people of Norwich have subscribed £10,000. The late Mr. Colman gave the site, Mrs. Reeve, wife of the High Sheriff, giving £1,000 to endow a cot to the memory of her daughters, and announced that her two grandchildren will maintain another cot in memory of their mother. At the meeting the Dean also announced another gift of £2,340; and one of £5,000 from Mr. Russell J. Colman and his family, in memory of his father. Contributions were also handed in from many others; and there now only remains to be collected the sum of £2,000 to secure the total estimated cost of £25,000. The infirmary is to contain 40 beds.

